# Parent-adolescent conflict and problematic internet use among Chinese adolescents: the mediating role of depression and the moderating role of school climate

**DOI:** 10.1186/s40359-024-01781-y

**Published:** 2024-05-21

**Authors:** Jiarong Chen, Shengnan Li, Yangang Nie

**Affiliations:** 1 Department of Psychology & Psychological and Behavioral Research Center of Adolescent, School of Education, Guangzhou University, 230 Wai Huan Xi Road, Guangzhou Higher Education Mega Center, Guangzhou, 510006 China; 2 Center for Studies of Psychological Application, South China Normal University, Guangzhou, 510631 China

**Keywords:** Parent-adolescent conflict, Depression, School climate, Problematic internet use, Adolescent

## Abstract

**Background:**

Problematic Internet use (PIU) may lead adolescents to physical, emotional, social, or functional impairment due to the risky, excessive, or impulsive internet use manner. How do the experiences of adolescents influence them using the internet in a problematic manner? The answer to this question is the key to preventing and intervening PIU of adolescents. To address this question, we focus on the interactions among family (parent-adolescent conflict), school (school climate), and individual factors (PIU, depression), exploring the influence factors of PIU.

**Methods:**

A moderated mediation model was constructed to explore the relationship between variables. Using a two-wave longitudinal design with a six-month interval between timepoints, this study collected data from 801 Chinese adolescents (411 boys, *M*_*age*_ = 14.68) by questionnaires. Path analysis was employed to test the model and participants’ age, sex and baseline were controlled.

**Results:**

Parent-adolescent conflict at Time 1 (T1) was positively related to PIU at Time 2 (T2) in adolescents. Depression at T2 mediated the relationship between parent-adolescent conflict at T1 and PIU at T2. School climate at T2 significantly moderated the mediation effect of depression on the relationship between parent-adolescent conflict at T1 and PIU at T2. Specifically, positive school climate could significantly weaken the negative effect of depression on PIU for adolescents with low level of depression.

**Conclusions:**

The present study reveals that parent-adolescent conflict leads to PIU in adolescents through depression whilst the school climate moderates the impacts of depression on PIU. This adds further evidence regarding the significance of systematically and consistently incorporating family and school in the alleviating of problem behaviors displayed by teens.

## Introduction

The Internet has become an indispensable part of people’s daily lives. According to the 51^st^ statistical report on China’s Internet development, the number of Internet users in China has been rapidly increasing, and reached 1.067 billion by the end of 2022. Moreover, the proportion of adolescent Internet users cannot be ignored, as 14.3% of all users are aged 10–19 years old [1]. While the Internet brings convenience to adolescents’ studies and lives [2], it also has severe consequences when used in a problematic manner. Problematic Internet use (PIU) refers to using the Internet in a risky, excessive, or impulsive manner, leading to adverse consequences, including physical, emotional, social, or functional impairment [3]. Several studies have demonstrated that PIU can lead negative psychological and behavioral outcomes to adolescents (e.g., depression, cyber aggression and victimization) [4–6]. Thus, exploring the risk factors associated with PIU and its underlying mechanism has both theoretical and practical importance for PIU prevention and intervention among adolescents.

Family environment as the prime ecological system has profound impacts on individuals’ development [7]. A negative family environment contributes to internalizing and externalizing problems in adolescents [8]. Previous cross-sectional studies have demonstrated that negative parent-child interaction (e.g., parent-child conflict) can directly lead to PIU in adolescents [9, 10]. However, cross-sectional studies could not prove the effect of parent-adolescent conflict on PIU over time [8, 11]. Therefore, the first aim of this study is to preliminarily explore the relationship between prior parent-adolescent conflict and subsequent PIU by a longitudinal method. According to the cognitive-affective personality system, situational features (e.g., parent-child conflict) could be encoded by the mediating cognitive and affective units (e.g., depression), generating distinctive behavior (e.g., PIU) in response to different situations [12]. But the cause of PIU as well as the intrinsic psychological mechanism between parent-adolescent conflict and PIU have not been well elucidated so far. Thus, the second aim of this study is to explore the mediating effect of depression between parent-adolescent conflict and PIU longitudinally. Individual behaviors are shaped through interactions in not only the family system but also the school environment [7]. As another main environment in which adolescents study and live, schools have a significant influence on their development [13]. A positive school climate could likely buffer the negative effect of parent-adolescent conflict and depression. Hence, the third aim of this study is to explore the protective effect of school climate on adolescent PIU.

### Parent-adolescent conflict and problematic internet use

As a type of negative parent-child interaction, parent-child conflict manifests as psychological or behavioral conflict between parent and child due to their opposition of cognition, emotion, behavior or attitude [14]. During puberty, adolescents tend to experience conflict with their parents when struggling to assert their autonomy and individuation [15]. Frequent and intense parent-adolescent conflict is harmful to adolescent development [16]. According to the uses and gratifications theory, individuals will use a medium (e.g., Internet, smartphones) to gratify their social needs [17, 18]. Parent-adolescent conflict not only prevents adolescents from meeting their increasing need to develop autonomy and individuation but also affects adolescents’ emotional insecurity and insecure attachment [19]. Therefore, adolescents may rely on Internet use to meet their unfulfilled social and psychological needs left unmet by their families [20, 21]. Moreover, parent-adolescent conflict is a main source of stress in the early stage of individual development [14]. Internet provides adolescents with a virtual environment in which they can indulge themselves without real-world stress [22, 23]. Previous cross-sectional researches have shown a positive relationship between parent-adolescent conflict and PIU [21, 24]. In longitudinal study, this association may still exist.

Based on the abovementioned theoretical and empirical evidence, we posit the first hypothesis:

#### Hypothesis 1

Parent-adolescent conflict is positively related to PIU in adolescents.

### The mediation effect of depression

Adolescents exposed to continuous parent-adolescent conflict would be sensitive to this kind of family stressors [25, 26], and developed depression as a response to these stressors [27, 28]. Empirical studies have demonstrated that both disagreement and hostility, which are two specific dimensions of parent-adolescent conflict, have negative effects on adolescent development [29, 30]. In addition, research has shown a strong direct association between parent-adolescent conflict and depression [31, 32]. To relieve negative emotions, adolescents with depression may turn to increase Internet use to enhance their mood and compensate for unmet needs caused by parent-adolescent conflict [20, 23, 33]. In addition, depression can deplete adolescents’ self-control resources, leading to a lack of self-control [34]. A previous study found that individuals with lower level of self-control will exhibit PIU more frequently [35].

Therefore, adolescents experiencing parent-adolescent conflict are likely to experience depression, leading to PIU. Accordingly, we propose the following hypothesis:

#### Hypothesis 2

Depression mediates the relationship between parent-adolescent conflict and PIU.

### The moderation effect of school climate

In this study, we paid close attention to the causes of PIU among adolescents and hoped to explore a moderating factor to help adolescents effectively intervene their PIU. Thus, guided by the ecological system theory [7], we focused on the moderating role of a positive school factor (e.g. school climate) in the pathway from parent-adolescent conflict and depression to PIU.

Although adolescents experiencing depression and parent-adolescent conflict may engage in PIU, they may not all experience negative consequences. School climate refers to a school’s quality and characteristics, and reflects school norms, values, goals, and interpersonal relationship [36]. According to stage environment fit theory, when school environment granted opportunity for adolescents, which fits their psychological needs, it wound boost their optimal development [37–39]. A positive school climate may provide interpersonal emotional support to adolescents with depression [40], thereby compensating for the lack of psychological need fulfillment caused by parent-adolescent conflict, which may reduce their PIU. Moreover, in a positive school climate, adolescents with depression may learn more appropriate Internet use, norms, and attitudes by observing their peers or teachers [41]. Thus, based on the evidence above, we propose the following:

#### Hypothesis 3

School climate moderates the mediating effect of depression on the relationship between parent-adolescent conflict and PIU, manifested as moderating the pathways from parent-adolescent conflict to PIU and from depression to PIU.

### The current study

**Fig. 1:**
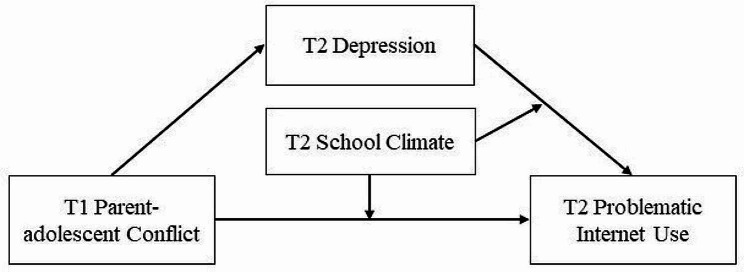
The proposed moderated mediation model

As literature illustrates, the previous cross-sectional studies failed to uncover the effect of parent-adolescent conflict on PIU over time and the potential psychological mechanism between the two variables. In this sense, it is meaningful to address the aforementioned research gaps for better understanding the causes of PIU and proposing effective intervention strategies for adolescents. Guided by the cognitive-affective personality system and the ecological system theory [7, 12], we concentrated on the interactions among family (parent-adolescent conflict), school (school climate), and individual emotional factors (depression), aiming at exploring the influencing factors of PIU. Hence, we constructed a moderated mediation model (Fig. 1) and adopted a two-wave longitudinal design to verify our hypotheses as follows: (1) Hypothesis 1: parent-adolescent conflict at T1 will be positively related to PIU in adolescents at both T1 and T2; (2) Hypothesis 2: T2 depression will mediate the relationship between T1 parent-adolescent conflict and T2 PIU, and (3) Hypothesis 3: T2 school climate will moderate the mediating effect of T2 depression on the relationship between T1 parent-adolescent conflict and T2 PIU, moderating the pathways from T1 parent-adolescent conflict to T2 PIU and from T2 depression to T2 PIU.

## Methods

### Participants and procedure

Convenience sampling was used to recruit participants from three junior and high schools in Guangzhou, China. Data were collected in two waves six months apart: November 2021 (T1) and May 2022 (T2). To examine the unidirectional impact of parent-adolescent conflict on PIU over time and the potential psychological mechanism, participants are required to report parent-adolescent conflict at T1, and depression and school climate at T2. Specifically, PIU was measured at both T1 and T2 to control the baseline of PIU. 881 participants finished the questionnaires at T1. Excluding participants who did not complete the measures at T2 and answer the questions seriously, the final sample included 801 adolescents, comprising 411 boys (51.3%) and 390 girls (48.7%) aged 12–17 years (14.68 ± 1.54 years).

The survey material and all procedures conformed to the Declaration of Helsinki and were approved by the Ethics Committee of Guangzhou University. In addition, the survey was conducted by well-trained data collectors. Before the formal survey, we obtained informed consent from all participants and their parents included in the study. Participants were informed that their responses would be strictly confidential and that they could opt out of the survey at any time if they became uncomfortable.

### Measures

#### Parent-adolescent conflict

The Parent-Child Conflict Scale designed by Fang et al. [42] was used to assess parent-child conflict among adolescents. The scale consists of eight items, requiring participants to report the frequency of conflicts with their parents in eight aspects (e.g., academic, making friends, spending money) and is rated on a five-point Likert scale ranging from 1 (never) to 5 (several times a day). In this study, Cronbach’s α was 0.85 for this scale. The result of confirmatory factor analysis (CFA) indicates the scale has acceptable structure validity: *χ*^*2*^*/df* = 3.98, CFI = 0.95, TLI = 0.93, RMSEA = 0.06, and SRMR = 0.04.

#### Depression

The Center for Epidemiologic Studies Depression Scale (CES-D) developed by Andresen et al. [43] was used to measure depression. The scale includes 10 items (e.g., “I find it hard to be concentrated”; “I feel very depressed”), ranging from 0 (never) to 3 (always). In this study, Cronbach’s α was 0.87 for this scale. The result of CFA indicates the scale has acceptable structure validity: *χ*^*2*^*/df* = 4.31, CFI = 0.96, TLI = 0.94, RMSEA = 0.06, and SRMR = 0.03.

#### School climate

The Delaware School Climate Surveys-Student Scale (DSCS-S) was used to measure school climate [44]. The scale comprises 29 total items across seven subscales: teacher-student relationship, student-student relationship, school-wide student engagement, clarity of expectations, fairness of rules, school safety, and school-wide bullying. Items are rated on a 4-point scale ranging from 1 (strongly disagree) to 4 (strongly agree). Sample items are “The school rules are fair” and “Teachers care about their students”. In this study, Cronbach’s α was 0.95 for this scale. The result of CFA indicates the scale has acceptable structure validity: *χ*^*2*^*/df* = 3.08, CFI = 0.91, TLI = 0.90, RMSEA = 0.05, and SRMR = 0.05.

#### Problematic internet use

The Chinese version of Young’s [45] Problematic Internet Use Scale was used to measure PIU. The scale comprises 10 items (e.g., Do you feel the need to use the internet with increasing amounts of time in order to achieve satisfaction) ranging from 1 (not at all true) to 6 (very true). In this study, Cronbach’s α was 0.90 at T1 and 0.91 at T2 for this scale. The result of CFA indicates the scale has acceptable structure validity at both T1 (*χ*^*2*^*/df* = 4.21, CFI = 0.95, TLI = 0.94, RMSEA = 0.06, SRMR = 0.03) and T2 (*χ*^*2*^*/df* = 4.24, CFI = 0.95, TLI = 0.94, RMSEA = 0.06, SRMR = 0.03).

### Statistical analyses

First, to ensure that the consistence and reliability of measurement tool across different time points, measurement invariance for PIU scores at two time points was evaluated using CFA. Following researchers’ recommendations, change in comparative fit index (ΔCFI) of more than 0.01 and change in root mean squared error of approximation (ΔRMSEA) of more than 0.015 would not able to support measurement invariance [46, 47].

Second, descriptive statistics and Pearson correlations were calculated by SPSS 22.0. We then used Mplus 8.3 [48] to test the moderated mediation effects through path analysis. Acceptable standard indices (χ^2^/*df* < 5, CFI and TLI > 0.90, RMSEA and SRMR < 0.08) were used to evaluate model fit following Hoyle [49]. Bootstrapping analysis with 5000 replicates was performed to verify the significance of the paths. For the path analysis, we set participants’ sex, age, and baseline (PIU at T1) as covariates in the longitudinal structural equation model to exclude their effects on the model outcome. Sex was dummy coded (0 = female, 1 = male).

## Results

### Common method bias

Considering that the questionnaires were self-reported by adolescents, common method bias could interfere with the results [50, 51]. To reduce the impact of common method bias in terms of procedure control, we emphasized the strict confidentiality of data to the participants and reverse-scored several items. Moreover, we employed Harman’s single-factor test to determine whether serious common method bias was present. The results showed that the eigenvalues of the 12 factors were > 1, and the first factor explained only 19.67% of the variance. Thus, the results indicated that no serious common method bias was present in this study.

### Preliminary analyses

The results of CFA suggested that the null hypothesis of measurement invariance should not be rejected (see Table 1). Table 2 displays the means, standard deviations, and Pearson correlations of the study variables. T1 parent-adolescent conflict was significantly positively correlated with T2 depression, T1 PIU, and T2 PIU, but significantly negatively correlated with T2 school climate. Moreover, T2 depression was significantly positively correlated with T1 and T2 PIU, but significantly negatively correlated with T2 school climate. T2 school climate was significantly negatively correlated with both T1 and T2 PIU. These results explain the relationship among the variables to a certain extent. However, the specific variable model requires further testing.

**Table 1:** Model fit statistics for tests of measurement invariance of PIU

Model Tested	χ^2^	df	CFI	RMSEA	TLI	Δ CFI	Δ RMSEA
Configural invariance	585.139	159	0.933	0.058	0.920		
Weak invariance	617.429	165	0.929	0.059	0.918	−0.004	+ 0.001
Strong invariance	618.937	174	0.930	0.057	0.924	+ 0.001	−0.002
Strict invariance	678.413	184	0.922	0.058	0.920	−0.008	+ 0.001

Note: PIU, Problematic Internet use;

**Table 2:** Descriptive statistics and correlations for all variables

Variable	M	SD	1	2	3	4	5	6
1. Sex	0.50	0.50						
2. Age	14.68	1.54	0.08^*^					
3. T1 PAC	1.78	0.75	−0.08^*^	−0.28^***^				
4. T2 DP	2.00	0.62	−0.20^***^	0.06	0.26^***^			
5. T2 SC	3.13	0.47	−0.04	−0.17^***^	−0.17^***^	−0.41^***^		
6. T1 PIU	2.68	1.06	0.00	0.17^***^	0.24^***^	0.41^***^	−0.30^***^	
7. T2 PIU	2.97	1.10	−0.06	0.18^***^	0.15^***^	0.36^***^	−0.28^***^	0.56^***^

Note: Sex was dummy-coded: 1 = male, 0 = female; PAC, Parent-adolescent conflict; DP, Depression; PIU, Problematic Internet use; SC, School climate; T1 = Time 1, T2 = Time 2; ^*^p < 0.05, ^***^p < 0.001

### Testing for mediation effects of depression

The paths from the covariates (i.e., age, sex, and T1 PIU) to all constructs were included as controls. However, the path between sex and T2 PIU was not statistically significant, and was therefore removed from the mediation model analysis to avoid overcontrol [52].

**Fig. 2:**
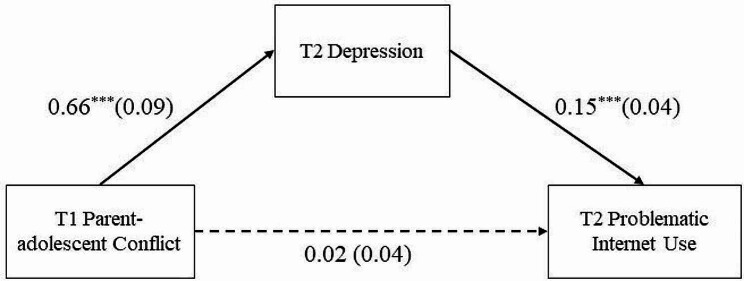
Model of the mediating role of T2 depression between T1 parent-adolescent conflict and T2 problematic Internet use Note: T1 = Time 1, T2 = Time 2. Values are standardized coefficients and the standard error. Paths of covariables in the model are not displayed. ^***^p < 0.001

The mediation model, as presented in Fig. 2, showed excellent data fit: *χ*^*2*^ = 1.93, *df* = 1, *χ*^*2*^*/df* = 1.93, CFI = 0.99, TLI = 0.99, and RMSEA = 0.03. As shown in Table 3; Fig. 2, after controlling for covariates, T1 parent-adolescent conflict positively predicted T2 depression (*β* = 0.66, *SE* = 0.09, *p* < 0.001, 95% CI = [0.51, 0.85]) but did not significantly positively predict T2 PIU (*β* = 0.02, *SE* = 0.04, *p* > 0.05, 95% CI = [− 0.05, 0.09]). T2 Depression positively predicted T2 PIU (*β* = 0.15, *SE* = 0.04, *p* < 0.001, 95% CI = [0.08, 0.22]).

**Table 3:** Mediation and moderated mediation effect test

Outcome variable	Predictive variable	*χ* ^*2*^ */df*	CFI	TLI	RMSEA	*β*	*SE*	*t*
**Mediation model**								
T2 DP	Age	1.93	0.99	0.99	0.03	1.49	0.24	6.10^***^
	Sex					−0.27	0.06	−4.62^***^
	T1 PAC					0.66	0.09	7.68^***^
T2 PIU	Age					0.10	0.03	3.16^**^
	T1 PIU					0.48	0.04	13.20^***^
	T1 PAC					0.02	0.04	0.43
	T2 DP					0.15	0.04	4.00^***^
**Moderated mediation model**								
T2 DP	Age	4.36	0.94	0.92	0.07	1.32	0.34	3.86^***^
	Sex					2.95	2.45	1.21
	T1 PAC					0.75	0.12	6.24^***^
T2 PIU	Age					0.08	0.03	2.65^**^
	Sex					−0.07	0.06	−1.30
	T1 PIU					0.46	0.04	12.80^***^
	T1 PAC					0.01	0.04	0.21
	T2 DP					0.13	0.04	3.34^**^
	T2 SC					−0.09	0.03	−2.53^*^
	T1 PAC × T2 SC					−0.02	0.04	−0.57
	T2 DP × T2 SC					0.11	0.05	2.32^*^

Note: All variables in the model have been standardized. Sex was dummy-coded: 1 = male, 0 = female; PAC, Parent-adolescent conflict; DP, Depression; PIU, Problematic Internet use; SC, School climate; T1 = Time 1, T2 = Time 2; ^*^p < 0.05, ^**^p < 0.01, ^***^p < 0.001

Additionally, bootstrapping analyses indicated that T2 depression mediated the relationship between T1 parent-adolescent conflict and T2 PIU (indirect effect = 0.10, *SE* = 0.03, *p* < 0.001, 95% CI = [0.05, 0.16]; see Table 4).

**Table 4:** Summary of the direct and indirect effects

Direct and indirect effects	Bias-corrected bootstrap estimated for the effects		
	β	SE	95%
**Direct pathway**			
T1 PAC → T2 PIU	0.02	0.04	[− 0.05, 0.09]
**Indirect pathway**			
T1 PAC → T2 DP → T2 PIU	**0.10**	**0.03**	**[0.05, 0.16]**

Note: PAC, Parent-adolescent conflict; DP, Depression; PIU, Problematic Internet use; T1 = Time 1; T2 = Time 2; Values are standardized. The significant results are in bold

### Testing for moderated mediation effect

**Fig. 3:**
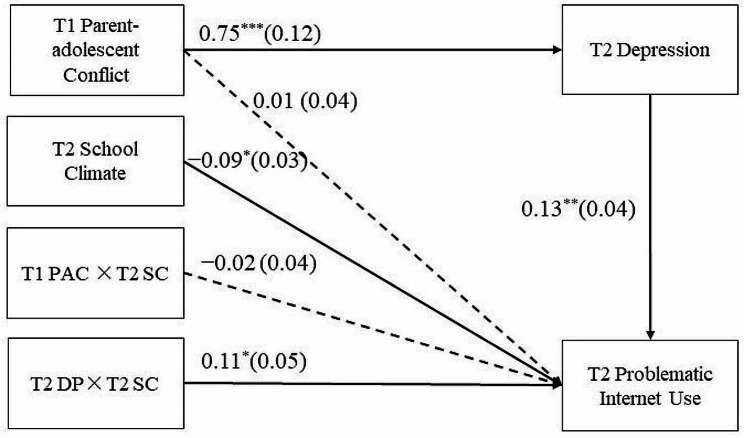
Model of the moderating role of T2 school climate on the indirect relation between T1 parent-adolescent conflict and T2 problematic Internet use via T2 depression Note: Values are standardized coefficients and the standard error. Paths of covariables in the model are not displayed. PAC, Parent-adolescent conflict; DP, Depression; SC, School climate; T1 = Time 1, T2 = Time 2. * *p* < 0.05, ** *p* < 0.01, *** *p* < 0.001

The moderated mediation model represented in Fig. 3 displays a good fit to the data: χ^*2*^ = 52.31, *df* = 12, *χ*^*2*^*/df* = 4.36, CFI = 0.94, TLI = 0.92, and RMSEA = 0.07. The bias-corrected percentile bootstrap results indicated that the indirect effect of T1 parent-adolescent conflict on T2 PIU through T2 depression was moderated by the T2 school climate.

Specifically, as shown in Table 2, T2 school climate moderated the relationship between T2 depression and T2 PIU (*β* = 0.11, *SE* = 0.05, *p* = 0.02, 95% CI = [0.02, 0.20]). Age, sex, and T1 PIU were included as covariates in the regression equation as controls. However, T2 school climate did not moderate the relationship between T1 parent-adolescent conflict and T2 PIU (*β* = −0.02, *SE* = 0.04, *p* = 0.57, 95% CI = [− 0.10, 0.05]).

**Fig. 4:**
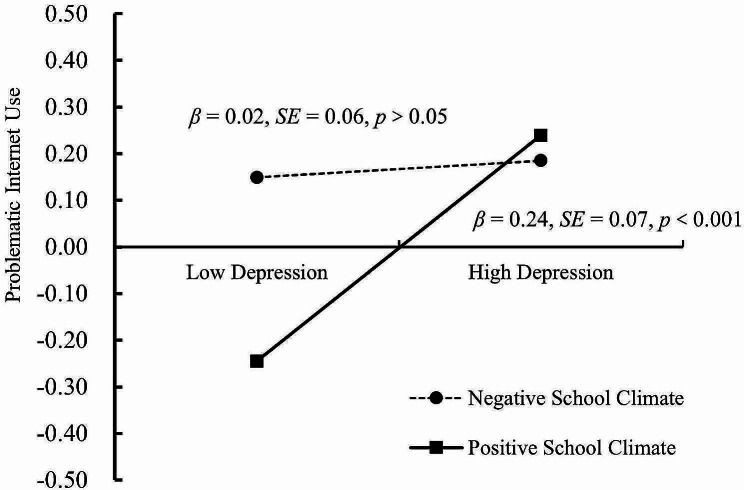
School climate as a protective function of depression and problematic internet use

To better understand the intrinsic mechanism of moderation, we conducted a simple slope test. As Fig. 4 shows, the positive link between T2 depression and T2 PIU was much weaker for adolescents with low school climate scores (1 *SD* below the mean; *β* = 0.02, *SE* = 0.06, *p* = 0.77, 95% CI = [− 0.10, 0.13]) than for those with a high school climate score (1 *SD* above the mean; *β* = 0.24, *SE* = 0.07, *p* < 0.001, and 95% CI = [0.12, 0.38]). Notably, the positive indirect links between T1 parent-adolescent conflict and T2 PIU via T2 depression were much weaker for adolescents with low school climate scores (indirect effect = 0.01, *SE* = 0.04, *p* = 0.77, 95% CI [− 0.07, 0.10]) than for those with high school climate scores (indirect effect = 0.18, *SE* = 0.06, *p* = 0.001, 95% CI = [0.09, 0.33]).

## Discussion

In the current study, we explored the relationship between parent-adolescent conflict and PIU longitudinally, as well as its underlying psychological mechanism in Chinese adolescents. The study yields three major findings. These findings have both theoretical and practical value in terms of preventing or providing interventions for PIU among adolescents.

### The association between parent-adolescent conflict and problematic internet use

Supporting hypothesis 1, we found parent-adolescent conflict was positively related to PIU in adolescents over time, which supplements longitudinal research evidence for the negative effect of parent-adolescent conflict on PIU. In line with the uses and gratifications theory [17, 18], this finding suggests that adolescents who experience more parent-adolescent conflicts are more prone to engage in Internet use in a risky, excessive, or impulsive manner to gratify their unmet social and psychological needs within their family [24]. Previous studies have found that the parent-adolescent relationship is a predictive factor for PIU [9, 53]. However, in this study, we did not find a direct predictive relation between T1 parent-adolescent conflict and T2 PIU. This may be due to the statistical control for the baseline level of T1 PIU, which is a strong predictor of subsequent PIU in adolescents. This suggests that parent-adolescent conflict is not sufficiently powerful to directly affect subsequent PIU when previous PIU is excluded. However, this does not mean that no association exists between parent-adolescent conflict and subsequent PIU. As our findings shows, parent-adolescent conflict will indirectly affect subsequent PIU via depression.

### The mediation of depression

Supporting Hypothesis 2, we found that depression mediated the relationship between parent-adolescent conflict and PIU, suggesting that prior parent-adolescent conflict would indirectly affect adolescents’ subsequent PIU by first influencing their subsequent depression. We found that parent-adolescent conflict was positively associated with depression. According to stress sensitization theory and life stress theory [25, 26], for adolescents, parent-adolescent conflict are not only early adverse environmental events but also later stressors. Adolescents who are exposed to either early or later parent-adolescent conflict may be at risk for experiencing depression symptoms and heavy stress, with this risk higher for those who experience both early and later parent-adolescent conflict [27, 28].

Further, depression was positively associated with PIU, which is consistent with the results of previous studies [54, 55]. Internet use can help adolescents satisfy their needs that are unmet in their real lives [56] and help them regulate negative moods [57]. Thus, the enjoyment that Internet use brings is likely to ultimately leading to PIU in adolescents with depression. According to the cognitive-behavioral model of PIU [58], individuals with depression are predisposed to develop maladaptive cognitions regarding the Internet, which subsequently leads to maladaptive Internet behaviors. Depression may also impair self-control [59], and individuals with poor self-control are more likely to engage in PIU [60]. In summary, depression not only increases individuals’ Internet use but also decreases their ability of using Internet appropriately, which will gradually create a self-perpetuating cycle, resulting in the onset of PIU [61].

### The moderation of school climate

Another important finding of the current study was that the mediating effect of depression was moderated by school climate, which supports Hypothesis 3. Specifically, school climate moderated the second stage of the mediating path (from depression to PIU). The relationship between depression and PIU was stronger for adolescents with positive school climates than for those with negative school climates. A positive school climate can buffer the negative impact of low level of depression on PIU. Social support brings adolescents love and care, providing them with sufficient psychological resources to relieve their acute psychological stress [62, 63]. In accordance with stage environment fit theory, a positive school climate provides adolescents with psychological support [64], thereby effectively reducing PIU in adolescents with low level of depression. Notably, a positive school climate was not found to affect adolescents with high level of depression, which supports the protective-reactive model [65]. As a protective factor, a positive school climate can diminish but not eliminate the association between the risk (depression) and the outcome (PIU). Adolescents with higher level of depression have more negative mood states [66], stress [67] and loss of self-control [68], which cannot be completely buffered by school climate and will directly lead to more serious PIU.

However, inconsistent with our hypothesis 3, T2 school climate did not moderate the relationship between T1 parent-adolescent conflict and T2 PIU. One possible explanation for this is that parent-adolescent conflict leads to individual negative externalizing behavior by influencing internalizing emotions [69]. Positive factors such as school climate cannot directly buffer problematic behaviors caused by negative family interactions but can alleviate PIU by regulating adolescents’ emotions. Meanwhile, many internalizing emotions can arise simultaneously due to parent-adolescent conflict [30]. Therefore, focusing interventions on an important emotional variable (depression) may be more effective than focusing on multiple emotional variables.

## Limitations and implications

This study has some limitations that need to be addressed in future research. First, we only explored the unidirectional impact of parent–adolescent conflict on PIU. Previous studies have demonstrated the negative effects of PIU on parent-adolescent conflict and depression [24, 70]. Future research can employ a cross-lagged design to further reveal the reciprocal relationship among parent-adolescent conflict, depression, and PIU. Second, we conducted our research on Chinese adolescents, which limits the generalizability of the results to different cultural samples. Future research to replicate our results should include participants from other cultural contexts. Third, although we used a longitudinal study design to examine our model, we only followed the participants for six months. This length of time is insufficient to observe the development of and changes in parent-adolescent conflict and its effects on PIU over time. Future research should extend the timeline to further explore changes with age.

However, the research findings also have important theoretical and practical implications. The study findings contribute to the understanding of the causes of PIU, validating cognitive-affective personality system as well as ecological system theory, and extending these theories to the knowledge on PIU. Meanwhile, these findings provide insights into integrating multiple theories when exploring the formation of problematic behaviors. As for practical implications for prevention and intervention in PIU, first, parents should learn to address issues with their children peacefully instead of through conflict. Second, parents should aim to better understand the characteristics and needs of adolescents during puberty to promote parent-child interactions and relationship. Third, if conflict does occur, we advise that parents address the problem calmly and express their love to their children in a timely manner to alleviate negative emotions of their children. Finally, schools need to create a safe, harmonious, and positive educational environment to effectively soothe adolescents’ emotions and positively guide and educate them to use the Internet appropriately.

## Conclusions

The present study reveals that parent-adolescent conflict leads to PIU in adolescents through depression whilst the school climate moderates the impacts of depression on PIU. This adds further evidence regarding the significance of systematically and consistently incorporating family and school in the alleviating of problem behaviors displayed by teens. With the prevalence of internet use among the young population, it is central that parents sustain a healthy relationship with their adolescent children and schools construct a secure and nurturing environment so as to lower students’ risks of the excessive and impulsive use of internet.

## Data Availability

The datasets used and analyzed during the current study are available from the corresponding author on reasonable request.

## Abbreviations

PIU: Problematic Internet use
CFI: Comparative Fit Index
RMSEA: Root-Mean-Square Error of Approximation
TLI: Tucker-Lewis Index
SRMR: Standardized Root Mean Square Residual

## References

[1] China Internet Network Information Center. The 51th China statistical report on Internet development. 2023. https://www.cnnic.net.cn/n4/2023/0303/c88-10757.html

[2] Dong H, Yang F, Lu X, Hao W (2020). Internet addiction and related psychological factors among children and adolescents in China during the coronavirus disease 2019 (COVID-19) epidemic. Front Psychiatry.

[3] Moreno MA, Jelenchick LA, Cheistakis DA (2013). Problematic internet use among older adolescents: a conceptual framework. Comput Hum Behav.

[4] Aboujaoude E (2010). Problematic internet use: an overview. World Psychiatry.

[5] Li S, Wang X, Wu Z, Zhang Y. The more internet access, the more mental symptoms students got, the more problematic internet use they suffered: a meta-analysis of mainland Chinese adolescents and young adults. Int J Ment Health Addict. 2022;1–21. 10.1007/s11469-022-00850-w 10.1007/s11469-022-00850-w PMC9244201 35789813

[6] Cebollero-Salinas A, Orejudo S, Cano-Escoriaza J, Íñiguez-Berrozpe T (2022). Cybergossip and problematic internet use in cyberaggression and cybervictimisation among adolescents. Comput Hum Behav.

[7] Bronfenbrenner U, Morris P, Lerner RM, Damon W (1998). The ecology of developmental processes. Handbook of child psychology: theoretical models of human development.

[8] Sela Y, Zach M, Amichay-Hamburger Y, Mishali M, Omer H (2020). Family environment and problematic internet use among adolescents: the mediating roles of depression and fear of missing out. Comput Hum Behav.

[9] Boniel-Nissim M, Sasson H (2018). Bullying victimization and poor relationships with parents as risk factors of problematic internet use in adolescence. Comput Hum Behav.

[10] Schneider LA, King DL, Delfabbro PH (2017). Family factors in adolescent problematic internet gaming: a systematic review. J Behav Addict.

[11] Li W, Garland EL, Howard MO (2014). Family factors in internet addiction among Chinese youth: a review of English- and chinese-language studies. Comput Hum Behav.

[12] Mischel W, Shoda Y (1995). A cognitive-affective system theory of personality: reconceptualizing situations, dispositions, dynamics, and invariance in personality structure. Psychol Rev.

[13] Crosnoe R, Benner AD, Bornstein MH, Leventhal T, Lerner RM (2015). Children at school. Handbook of child psychology and developmental science: ecological settings and processes.

[14] Xu Y, Zhou Y, Zhao J, Xuan Z, Li W, Han L, Liu H (2021). The relationship between shyness and aggression in late childhood: the multiple mediation effects of parent-child conflict and self-control. Pers Individ Differ.

[15] Branje S (2018). Development of parent-adolescent relationships: conflict interactions as a mechanism of change. Child Dev Perspect.

[16] Sun L, Ju J, Kang L, Bian Y (2021). More control, more conflicts? Clarifying the longitudinal relations between parental psychological control and parent-adolescent conflict by disentangling between-family effects from within-family effects. J Adolesc.

[17] Katz E, Blumler JG, Gurevitch M, Blumler JG, Katz E (1974). Utilization of mass communication by the individual. The uses of mass communications: current perspectives on gratifications research.

[18] Kim D, Nam JK, Oh J, Kang MC (2016). A latent profile analysis of the interplay between PC and smartphone in problematic internet use. Comput Hum Behav.

[19] Martin MJ, Sturge-Apple ML, Davies PT, Gutierrez G (2019). Attachment behavior and hostility as explanatory factors linking parent-adolescent conflict and adolescent adjustment. J Fam Psychol.

[20] Dou K, Feng XK, Wang LX, Li JB (2022). Longitudinal association between parental involvement and internet gaming disorder among Chinese adolescents: consideration of future consequences as a mediator and peer victimization as a moderator. J Behav Addict.

[21] King DL, Delfabbro PH (2017). Features of parent-child relationships in adolescents with internet gaming disorder. Int J Ment Health Addict.

[22] Song H, Zmyslinski-Seelig A, Kim J, Drent A, Victor A, Omori K, Allen M (2014). Does Facebook make you lonely? A meta-analysis. Comput Hum Behav.

[23] Wang L, Dou K, Li J, Zhang M, Guan J (2021). The association between interparental conflict and problematic internet use among Chinese adolescents: testing a moderated mediation model. Comput Hum Behav.

[24] Özaslan A, Yıldırım M, Güney E, Güzel HS, İşeri E (2022). Association between problematic internet use, quality of parent-adolescents relationship, conflicts, and mental health problems. Int J Ment Health Addict.

[25] Monroe SM, Harkness KL (2005). Life stress, the kindling hypothesis, and the recurrence of depression: considerations from a life stress perspective. Psychol Rev.

[26] Hammen C, Henry R, Daley SE (2000). Depression and Sensitization to Stressors among Young Women as a function of Childhood Adversity. J Consult Clin Psychol.

[27] Brown GW, Harris T (1979). Social origins of depression: a study of psychiatric disorder in women. Soc Forces.

[28] Gotlib IH, Hammen CL (2008). Handbook of depression.

[29] Lewis G, Collishaw S, Thapar A, Harold GT (2014). Parent-child hostility and child and adolescent depression symptoms: the direction of effects, role of genetic factors and gender. Eur Child Adolesc Psychiatry.

[30] Weymouth BB, Buehler C, Zhou N, Henson RA (2016). A meta-analysis of parent-adolescent conflict: disagreement, hostility, and youth maladjustment. J Fam Theory Rev.

[31] Samek DR, Wilson S, McGue M, Iacono WG (2018). Genetic and environmental influences on parent-child conflict and child depression through late adolescence. J Clin Child Adolesc Psychol.

[32] Smith OA, Nelson JA, Adelson MJ (2019). Interparental and parent-child conflict predicting adolescent depressive symptoms. J Child Fam Stud.

[33] Liang L, Zhou D, Yuan C, Shao A, Bian Y (2016). Gender differences in the relationship between internet addiction and depression: a cross-lagged study in Chinese adolescents. Comput Hum Behav.

[34] Qiu C, Liu Q, Yu C, Li Z, Nie Y (2022). The influence of meaning in life on children and adolescents’ problematic smartphone use: a three-wave multiple mediation model. Addict Behav.

[35] Kim J, Hong H, Lee J, Hyun MH (2017). Effects of time perspective and self-control on procrastination and internet addiction. J Behav Addict.

[36] Rudasill KM, Snyder KE, Levinson H, Adelson JL (2018). Systems view of school climate: a theoretical framework for research. Educ Psychol Rev.

[37] Burns EC (2020). Factors that support high school completion: a longitudinal examination of quality teacher-student relationships and intentions to graduate. J Adolesc.

[38] Eccles JS, Midgley C, Ames RE, Ames C (1989). Stage-environment fit: developmentally appropriate classrooms for young adolescents. Research on motivation in education.

[39] Eccles JS, Midgley C, Wigfield A (1993). Development during adolescence. The impact of stage-environment fit on young adolescents’ experiences in schools and in families. Am Psychol.

[40] Yu C, Li X, Wang S, Zhang W (2016). Teacher autonomy support reduces adolescent anxiety and depression: an 18-month longitudinal study. J Adolesc.

[41] Zhai B, Li D, Li X (2020). Perceived school climate and problematic internet use among adolescents: mediating roles of school belonging and depressive symptoms. Addict Behav.

[42] Fang X, Dong Q. Parent-adolescent conflicts during early adolescence. J Psychol Sci. 1998;2122–5. 10.16719/j.cnki.1671-6981.1998.02.007

[43] Andresen EM, Malmgren JA, Carter WB, Patrick DL (1994). Screening for depression in well older adults: evaluation of a short form of the CES-D (center for epidemiologic studies Depression Scale). Am J Prev Med.

[44] Bear GG, Gaskins C, Blank J, Chen FF (2011). Delaware school climate survey-student: its factor structure, concurrent validity, and reliability. J Sch Psychol.

[45] Young KS (1998). Internet addiction: the emergence of a new clinical disorder. Cyberpsychol Behav.

[46] Chen FF (2007). Sensitivity of goodness of fit indexes to lack of measurement invariance. Struct Equ Model.

[47] Cheung GW, Rensvold RB (2002). Evaluating goodness-of-fit indexes for testing measurement invariance. Struct Equ Model.

[48] Muthén LK, Muthén BO, Mplus (2017). Statistical analysis with latent variables: user’s guide (Version 8).

[49] Hoyle RH (2012). Handbook of structural equation modeling.

[50] Podsakoff PM, MacKenzie SB, Lee JY, Podsakoff NP (2003). Common method biases in behavioral research: a critical review of the literature and recommended remedies. J Appl Psychol.

[51] Wen Z, Huang B, Tang D (2018). Preliminary work for modeling questionnaire data. J Psychol Sci.

[52] Chen BB, Qu Y, Yang B, Chen X (2022). Chinese mothers’ parental burnout and adolescents’ internalizing and externalizing problems: the mediating role of maternal hostility. Dev Psychol.

[53] Wang W, Li D, Li X (2018). Parent-adolescent relationship and adolescent internet addiction: a moderated mediation model. Addict Behav.

[54] Yang X, Guo WJ, Tao YJ (2022). A bidirectional association between internet addiction and depression: a large-sample longitudinal study among Chinese university students. J Affect Disord.

[55] Zhang Y, Liu Z, Zhao Y (2021). Impulsivity, social support and depression are associated with latent profiles of internet addiction among male college freshmen. Front Psychiatry.

[56] Zhao Y, Qu D, Chen S, Chi X (2023). Network analysis of internet addiction and depression among Chinese college students during the COVID-19 pandemic: a longitudinal study. Comput Hum Behav.

[57] Li G, Hou G, Yang D, Jian H, Wang W (2019). Relationship between anxiety, depression, sex, obesity, and internet addiction in Chinese adolescents: a short-term longitudinal study. Addict Behav.

[58] Davis RA (2001). A cognitive-behavioral model of pathological internet use. Comput Hum Behav.

[59] Li JB, Delvecchio E, Lis A, Nie YG, Di Riso D (2015). Parental attachment, self-control, and depressive symptoms in Chinese and Italian adolescents: test of a mediation model. J Adolesc.

[60] Özdemir Y, Kuzucu Y, Ak Ş (2014). Depression, loneliness and internet addiction: how important is low self-control?. Comput Hum Behav.

[61] Moreno MA, Jelenchick LA, Breland DJ (2015). Exploring depression and problematic internet use among college females: a multisite study. Comput Hum Behav.

[62] Cohen S, Wills TA (1985). Stress, social support, and the buffering hypothesis. Psychol Bull.

[63] Ren X, Wang Y, Hu X, Yang J (2019). Social support buffers acute psychological stress in individuals with high interdependent self-construal. Acta Physiol Sinca.

[64] Dou K, Wang LX, Cheng DL, Li YY, Zhang MC (2022). Longitudinal association between poor parental supervision and risk-taking behavior: the role of self-control and school climate. J Adolesc.

[65] Fergus S, Zimmerman MA (2005). Adolescent resilience: a framework for understanding healthy development in the face of risk. Annu Rev Public Health.

[66] Ostovar S, Allahyar N, Aminpoor H, Moafian F, Md Nor MB, Griffiths MD (2016). Internet addiction and its psychosocial risks (depression, anxiety, stress and loneliness) among Iranian adolescents and young adults: a structural equation model in a cross-sectional study. Int J Ment Health Addict.

[67] Dulaney ES, Graupmann V, Grant KE, Adam EK, Chen E (2018). Taking on the stress-depression link: meaning as a resource in adolescence. J Adolesc.

[68] Yang X, Zhao J, Chen Y, Zu S, Zhao J (2018). Comprehensive self-control training benefits depressed college students: a six-month randomized controlled intervention trial. J Affect Disord.

[69] Thomas SA, Jain A, Wilson T (2019). Moderated mediation of the link between parent-adolescent conflict and adolescent risk-taking: the role of physiological regulation and hostile behavior in an experimentally controlled investigation. J Psychopathol Behav Assess.

[70] Chi X, Liu X, Guo T, Wu M, Chen X (2019). Internet addiction and depression in Chinese adolescents: a moderated mediation model. Front Psychiatry.

